# Food stoichiometry affects the outcome of *Daphnia*–parasite interaction

**DOI:** 10.1002/ece3.545

**Published:** 2013-04-02

**Authors:** Sanni L Aalto, Katja Pulkkinen

**Affiliations:** Department of Biological and Environmental Sciences, University of JyväskyläJyväskylä, Finland

**Keywords:** Ecological stoichiometry, host–parasite interaction, microsporidian, multiple stressors, P-deficiency

## Abstract

Phosphorus (P) is an essential nutrient for growth in consumers. P-limitation and parasite infection comprise one of the most common stressor pairs consumers confront in nature. We conducted a life-table study using a *Daphnia*–microsporidian parasite model, feeding uninfected or infected *Daphnia* with either P-sufficient or P-limited algae, and assessed the impact of the two stressors on life-history traits of the host. Both infection and P-limitation negatively affected some life-history traits tested. However, under P-limitation, infected animals had higher juvenile growth rate as compared with uninfected animals. All P-limited individuals died before maturation, regardless of infection. The numbers of spore clusters of the microsporidian parasite did not differ in P-limited or P-sufficient hosts. P-limitation, but not infection, decreased body phosphorus content and ingestion rates of *Daphnia* tested in separate experiments. As parasite spore production did not suffer even under extreme P-limitation, our results suggest that parasite was less limited by P than the host. We discuss possible interpretations concerning the stoichiometrical demands of parasite and suggest that our results are explained by parasite-driven changes in carbon (C) allocation of the hosts. We conclude that the impact of nutrient starvation and parasite infection on consumers depends not only on the stoichiometric demands of host but also those of the parasite.

## Introduction

Parasites are significant biotic stressors detrimentally affecting individuals and populations of host species. In contrast to other stressors (predation, competition, pesticides), parasites have a reciprocal relationship with the target organism, as parasites drain their energy from the host. Thus the presence of other stressors that negatively affect the host can also indirectly mediate the progress of infection or development of the parasite itself (Lafferty and Kuris 1999; Duffy et al. 2011). Subsequently, other stressors may alter the virulence of the parasite, that is, the fitness cost imposed on the host by the parasite (Jokela et al. 2005; Johnson et al. 2006a, 2007; Coors and De Meester 2008; Coors et al. 2008).

Host nutrition has an important role in host–parasite interactions. Previous studies have mainly concentrated on the effect of food quantity on host physiological functions (e.g., immune defense) and the development of the parasite infection. For example, food deprivation decreased parasite load in the water flea *Daphnia magna* (Pulkkinen and Ebert 2004) and in the snail *Lymnaea stagnalis* (Seppälä et al. 2011). Fewer studies have considered the effect of food quality on host–parasite interactions. In the caterpillar *Spodoptera exempta*, survival of bacterially infected larvae was lower with decreasing dietary protein-to-carbohydrate ratio (Povey et al. 2009). Recently, studies have (Frost et al. 2008; Hall et al. 2009) demonstrated poor nutritional condition (low food phosphorus P concentration) of the host to decrease parasite within-host reproduction.

Nutrient competition between the host and the parasite may determine the outcome of infection (Smith 1993) and could be expected to depend on the stoichiometric demands of both partners. If parasite and host are competing for the same elemental nutrient, parasite effects on host physiological status might be more drastic and it might thus express higher virulence than in a situation where stoichiometric demands of the parasite and the host differ. The strength of nutrient competition could be expected to vary among different host–parasite combinations.

Phosphorus is an important component for numerous key molecules, including nucleid acids (RNA and DNA) and energetic nucleotides (ATP) and as such, essential for organism growth and function (Sterner and Elser 2002). Consumers have high demand for P for optimal growth and reproduction (Elser et al. 2000; Hessen 2008). However, producer carbon:phosphorus (C:P) ratios vary widely following environmental nutrient concentrations (Sterner and Elser 2002; Sterner et al. 2008) and autotrophs with high C:P are common (Elser et al. 2000, 2010). This leads to a stoichiometric mismatch, where consumers (notably grazers) are limited by P which implicitly means C is in excess for (nearly) homeostatic consumers. Thus P-limitation will cause reduced growth efficiency in terms of C or energy, and it may consequently change the life-history parameters, behavior or physiological status of organisms (Urabe et al. 1997; Sterner and Elser 2002; Frost et al. 2005, 2010). Considering the frequency of P-limitation and ubiquity of parasites in nature, they constitute a powerful combination of mutual stressors.

**Figure 1:**
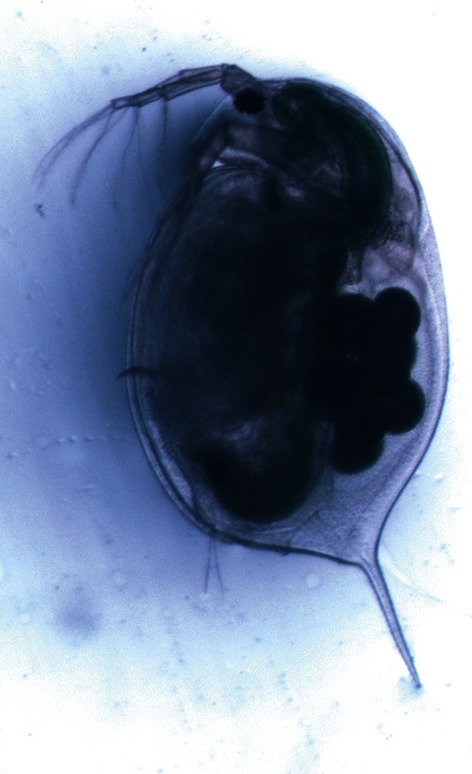
Water flea *Daphnia magna*.

In this study, we address the impact of P-limitation on parasite virulence and their combined effect on key life-history traits of host. The host, water flea *D. magna* (Fig. 1), has high requirements for nutritional phosphorus (∼1% P; Main et al. 1997) and P-limitation has major negative impact on growth and reproduction (Sundbom and Vrede 1997; Jeyasingh et al. 2011). The parasite, *Glugoides intestinalis*, is a microsporidian endoparasite, which infects host gut epithelial cells and thus shares host energy and nutrient supply (Ebert 1995; Larsson et al. 1996). It is rather avirulent (Ebert et al. 2000) as compared with parasite species used in previous studies concerning P-limitation and parasite infection (Frost et al. 2008; Hall et al. 2009).

We conducted a life-table experiment, feeding uninfected or infected *Daphnia* either with P-sufficient or P-limited algae. Key life-history traits were recorded over one life cycle in order to see at which stage P-limitation alters parasite virulence. We also followed the progress of the parasite infection in order to estimate the growth of the parasite. On the basis of earlier knowledge (Hessen et al. 2002; DeMott 2003), we expected strong negative effects of P-limitation on *Daphnia* survival, growth, and reproduction. Similarly, we expected negative fitness effects on hosts fed P-sufficient food when infected (Ebert et al. 2000). Regarding the interaction between P-limitation and infection, we considered two alternative outcomes. If the parasite had high requirements for P, parasite spore production and its virulence would decrease in the P-limited host. However, if the parasite was less limited by P than the *Daphnia* host, parasite would be able to grow and even increase in virulence under P-limitation.

## Materials and Methods

### Study system

The model used in the experiment consisted of a freshwater crustacean *D. magna* Straus (Crustacea: Cladocera) and its obligatory parasitic microsporidian *G. intestinalis* Chatton (Microspora: Glugeidea; Larsson et al. 1996). *G. intestinalis* is an intracellular parasite infecting host's gut epithelial cells through waterborne spores. It reproduces directly and transmission is horizontal between hosts (Ebert 1995). Hosts do not recover from infection (Ebert et al. 2000). *G. intestinalis* produces spherical clusters of 20–30 spores (Ebert and Mangin 1997). The number of clusters increases exponentially during the early infection (by more than one order of magnitude from day 7 to day 13 after infection; Ebert 1994, 1995). The *Daphnia* clone (DK-35-9) used in the experiment has been maintained in the laboratory for several years. It is the original host for the parasite *G. intestinalis*.

### Life-table experiment

The food algae, *Scenedesmus gracilis*, was grown in semibatch cultures in modified WC medium (Guillard and Lorenzen 1972, without vitamin solution) with a biweekly renewal corresponding to a dilution rate 0.2 per day. P-sufficient algae was grown in medium containing 50 μmol P/L, yielding a stable C:P ratio of approximately 200. For P-limited algae, the P-concentration was reduced to 5 μmol P/L, which resulted in C:P ratios ranging between 900 and 1100. The cultures were grown at least 2 weeks before starting the experiment to attain steady state. The cell density was calculated for each batch of algae used for feeding. As a proxy of C content we used cell numbers and C concentrations (mg C per cell) from a preliminary algae growth experiment (S. L. Aalto and K. Pulkkinen unpubl. data).

Prior to the experiment, *Daphnia* females were transferred to glass jars filled with 200 mL ADaM (Klüttgen et al. 1994; modified by using only one twentieth of the SeO_2_ concentration) in groups of 10–20 animals. They were fed ad libitum with a suspension of the P-sufficient food algae. Experiments were started with neonates from at least second brood of these mothers born within 12 h. Neonates were distributed randomly in groups of 10 into 100 mL of ADaM. Half of the neonates were exposed to parasite infection by cohabitation with five *D. magna* females infected with *G. intestinalis* for 24 h. Control animals were treated similarly with females from uninfected cultures. During exposure, experimental animals were fed with P-sufficient algae at 2 mg C/L. After 24 h, animals were transferred individually into 50 mL of ADaM. The females used for infection were dissected and checked for presence of infection in order to verify exposure of all experimental animals in the infected treatment.

In the experiment, 48 replicates of infected and 20 of uninfected were fed with P-sufficient algae and 58 and 40, infected and uninfected, respectively, with P-limited algae. Of these, 25 randomly selected infected individuals fed with P-sufficient algae and 15 fed with P-limited algae were allocated only for following the progress of parasite infection (see later). Individuals in both treatments received 1 mg algal C/L per day on first 6 days and subsequently 2 mg C/L per day. Every other day, individuals were transferred with a pipette in a small volume of ADaM onto a glass slide and photographed with video camera (GO-5-CLR-12, QImaging, Surrey, Canada) attached to research stereo microscope (SZX9, Olympus, Hamburg, Germany). Then they were transferred to fresh media and fed. Juveniles released were counted and discarded.

The photographs were analyzed with ImageJ (Image Processing and Analysis in Java; version 1.44) to measure the length of *Daphnia* (top of the helmet to the base of the caudal spine) and to detect the eggs in brood chamber. Lengths were converted into dry weights using separate preestablished relationships for all four treatments. For P-sufficient treatment: Dry weight (μg) = 10.4* length (mm)^3.03^, *r*^2^ = 0.89, Dry weight (μg) = 8.4* length (mm)^2.28^, *r*^2^ = 0.78, for uninfected and infected animals, respectively. For P-limited treatment: Dry weight (μg) = 10.2* length (mm)^0.84^, *r*^2^ = 0.32, Dry weight (μg) = 9.9* length (mm)^1.39^, *r*^2^ = 0.54, for uninfected and infected animals, respectively.

Growth (*g*) was calculated from individual dry weights (*W*_1_ and *W*_2_) at successive times (*t*_1_ and *t*_2_) according to Lampert and Trubetskova (1996):


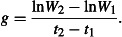


We calculated two different indices: juvenile growth (*g*_j_) was calculated as a difference in individual dry weight at day 6 (*W*_2_) and day 0 (*W*_1_). To calculate the growth until maturation (*g*_m_), *W*_2_ was defined individually as the day when first clutch of eggs were released into the brood pouch. In uninfected animals this age varied between 8 and 12 days and in infected between 10 and 16 days. Experiment was ended when remaining animals had produced their third clutch (day 26).

To follow the progress of parasite infection during the experiment, randomly chosen individuals fed with P-sufficient algae were dissected at ages of 13, 21, and 26 days. In addition, all remaining animals from the life-history experiment at day 26 were dissected. The first time point was chosen based on earlier knowledge that parasite infection is fully developed and spore clusters are visible inside host gut cells on days 10–13 after the establishment of the infection (Ebert 1994, 1995; K. Pulkkinen unpubl. data). Due to high mortality, animals fed with P-limited algae were dissected already at ages of 9 and 13 days and infection could not be followed further. Spore loads of the animals were determined from freshly dissected guts by enumerating the number of spore clusters within epithelial cells under microscope (Leitz Biomed, Leica Microsystems, Wetzlar, Germany) using 100–400 × magnification with phase contrast (Ebert and Mangin 1997).

In order to calculate C:P ratios of the algae, subsamples were collected and freeze dried (Alpha 1-4 LD Plus, Martin Christ Gefriertrocknungsanlagen GmbH, Osterode, Germany). Particulate C was analyzed on Carlo-Erba Flash 1112 series Elemental Analyser (Thermo Fisher Scientific, Waltham, MA). Particulate P was measured with QuickChem 8000 flow injection analysis system (LaChat instruments, Loveland, CO). To measure carbon per liter (mg C/L), subsamples of algae were filtered on preweighed CF/C filters, dried in 60°C and weighed with analytical balance (ED224S, Sartorius AG, Göttingen, Germany).

### Phosphorus content and ingestion rate of *Daphnia*

Neonates born within 24 h from at least second brood of mothers kept in standard conditions were distributed randomly in groups of 20 into 100 mL of ADaM and exposed either to infection or treated as uninfected controls as described above for the life-table experiment. Eleven replicates of infected and 11 replicates of uninfected animals (20 per replicate) were subsequently fed with P-sufficient algae, and 12 and 11, infected and uninfected, respectively, with P-limited algae.

Four replicates of each treatment were analyzed for body P content at the age of 6 days and the remaining replicates at age of 12 days. Some P-limited replicates suffered from high mortality, leaving fewer replicates for analyses at age of 12 days. Animals were rinsed with deionized water, collected into glass scintillation vials, and freeze dried. Samples were combusted (450°C, 4 h), diluted in 0.2N H_2_SO_4_, and analyzed with QuickChem 8000 analyzer.

To measure ingestion rate (1000 cells ind^−1^ h^−1^), nine replicates of infected and nine replicates of uninfected animals (ten per replicate) were fed with P-sufficient algae, and eight infected and eight uninfected replicates with P-limited algae. A batch of P-sufficient algae was radiolabelled with 1.5 MBq of carrier-free radioisotope ^33^P. After 48 h, the algae were centrifuged (1500 rpm, 5 min) to remove any dissolved radioactive isotope and resuspended into ADaM. Unlabeled subculture of algae was treated in the same way and analyzed for C:P ratios and algal cell concentrations as in the life-table experiment (see above). Ingestion rates were measured from four replicates of infected and four replicates of uninfected P-sufficient animals, and three infected and three uninfected replicates of P-limited animals at the age of 6 days and from the remaining replicates at the age of 12 days. In each ingestion trial, 1–3 animals were allowed to feed with labeled P-sufficient algae for 8 min, then rinsed with ADaM and transferred to scintillation vials. To measure algal and background activities, 2 mL of the feeding solution used in the ingestion trial as well as 2 mL of solution filtered through 0.2 μm Nucleopore filter was put in separate scintillation vials. The sample volume in *Daphnia* samples was adjusted to 2 mL with ADaM. Samples were solubilized with 1 mL of Solvable™ (Perkin Elmer, Waltham, MA) at room temperature overnight. Next day, activity of the ^33^P was determined with scintillation counter (Rackbeta, Perkin Elmer) using 10 mL of scintillation cocktail (HiSafe3, Perkin Elmer), channel 50–190 and counting time of 10 min, resulting in 100–3000 counts per minute for *Daphnia* and 1000–8000 for algae samples. Activity in filtrate samples was close to background values. Ingestion rate was calculated following Lampert and Taylor (1985).

### Statistical analyses

All statistical analyses were conducted using PASW **(**version 18.0, IBM Corporation, Armonk, NY). Two-way analysis of variance (two-way ANOVA) or three-way analysis of variance (three-way ANOVA) was used if the data met or could be transformed to meet the normality assumptions. Otherwise, nonparametric tests (Mann–Whitney *U*-test) were used. Differences in the survival of individuals during the experiment were analyzed with Cox regression analysis using a time-dependent covariate and interaction of food treatment and infection status as covariates, and food treatment and infection status as categorical covariates. Growth curves were analyzed with a nonlinear regression model. The model was based on von Bertalanffy growth equation for weight (*W*_age_ = *W*_max_ × (1−e^(−*K* × (age−t^_0_^))^)^3^), where *W*_max_ is the estimated asymptotic size, *K* is curvature index, and *t*_0_ is age of size 0. Curves with nonoverlapping confidence intervals of the asymptotic sizes were determined to be statistically significantly different.

## Results

**Figure 2:**
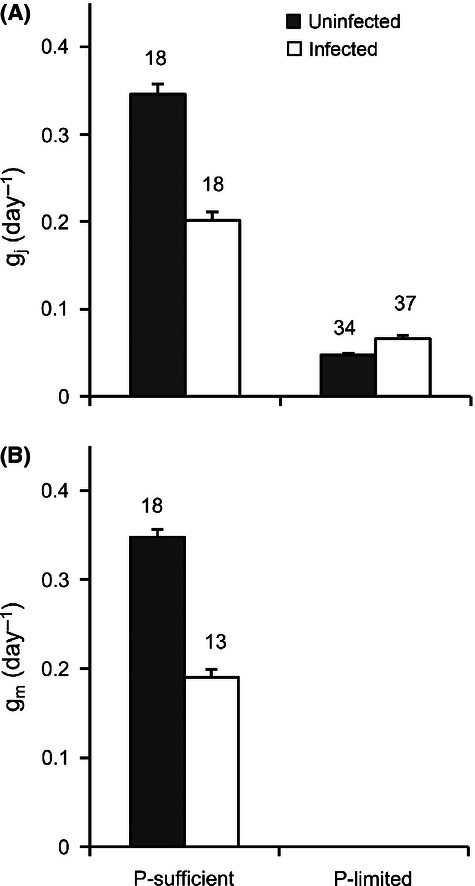
Mean (±SE) juvenile growth rate (*g*_j_) (A) and growth until maturation (*g*_m_) (B) in response to infection status and food quality. Sample sizes (number of *Daphnia* measured) are indicated above the bars.

P-limitation inhibited the growth of *Daphnia* at juvenile stage (*g*_j_) relative to P-sufficient animals (Fig. 2A). The interaction between food and infection was statistically significant (two-way ANOVA F_1,102_ = 172, *P* < 0.001), such that in P-limited treatment infected individuals had higher growth rate compared with uninfected ones (F_1,102_ = 36, *P* < 0.001), but vice versa in P-sufficient treatment (F_1,102_ = 140, *P* < 0.001). As all P-limited animals died before maturation, growth until maturation (*g*_m_) was calculated only for P-sufficient animals, in which the infection impaired the growth statistically significantly (two-way ANOVA, F_1,32_ = 96, *P* < 0.001, Fig. 2B). Infected maturing animals had lower specific growth rates than juvenile animals (paired *t*-test, *t* = 3.1, *P* = 0.008), but there was no difference within uninfected animals (paired *t*-test, *t* = −0.4, *P* = 0.68).

**Figure 3:**
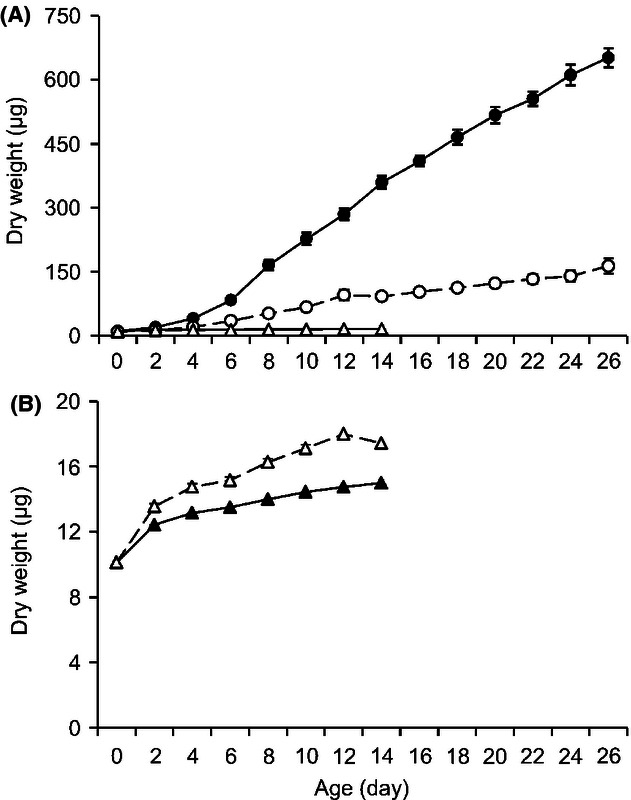
The growth of *Daphnia magna* over time for P-sufficient (filled dots), infected P-sufficient (open dots), P-limited (filled triangles), and infected P-limited (open triangles) treatments (A). Due to the large scale in panel (A) hiding the difference between the P-limited treatments, the growth curves for P-limited (filled triangles) and infected P-limited (open triangles) are presented (B). Means ± SE are depicted.

These results were further confirmed by nonlinear regression models for the growth curves, which showed that the asymptotic weight of individuals fed with P-limited food (17 ± 3 μg ind^−1^; mean ± 95% confidence intervals) was substantially lower than those fed with P-sufficient food (1046 ± 666 μg ind^−1^; Fig. 3). The asymptotic weight did not differ between uninfected and infected P-limited individuals (17 ± 2 μg and 20 ± 6 μg ind^−1^, respectively), while infected individuals in the P-sufficient treatment (195 ± 52 μg ind^−1^) reached lower weight than uninfected individuals (850 ± 70 μg ind^−1^).

**Figure 4:**
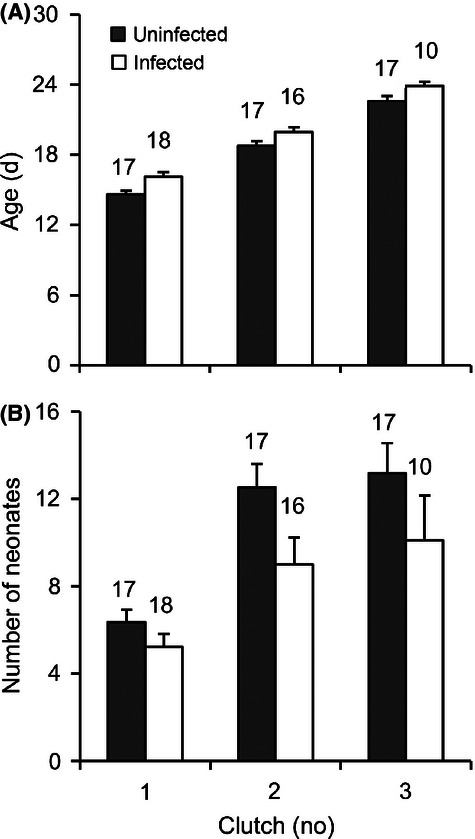
Mean (±SE) production age for each clutch (A) and size of the clutch for each clutch (B) in uninfected and infected P-sufficient individuals. Sample sizes (number of *Daphnia* measured) are indicated above the bars.

All P-limited animals died before maturation, thus we could compare reproductive parameters only between infected and uninfected *Daphnia* fed with P-sufficient food. Body mass at maturity was lower for infected (107 ± 3 μg) than for uninfected *Daphnia* females (365 ± 14 μg; ANOVA for sqrt-transformed values, F_1,34_ = 450, *P* < 0.001). Infected females produced their first brood later than uninfected females (Mann–Whitney *U*-test, *U* = 93, *P* = 0.029, Fig. 4A). Similar trend was seen within the next clutches (second clutch: *U* = 81, *P* = 0.05; third clutch: *U* = 41, *P* = 0.03). The average clutch size was smaller in infected animals, but the difference was not statistically significant within each clutch (*U* = 128, *P* = 0.29; *U* = 86, *P* = 0.11; *U* = 66, *P* = 0.36; for first, second, and third clutch, respectively, Fig. 4B). However, the cumulative number of neonates born during the experiment (of the first three clutches) was smaller in infected than in uninfected females (ANOVA, F_1,34_ = 9.93, *P* = 0.003).

**Figure 5:**
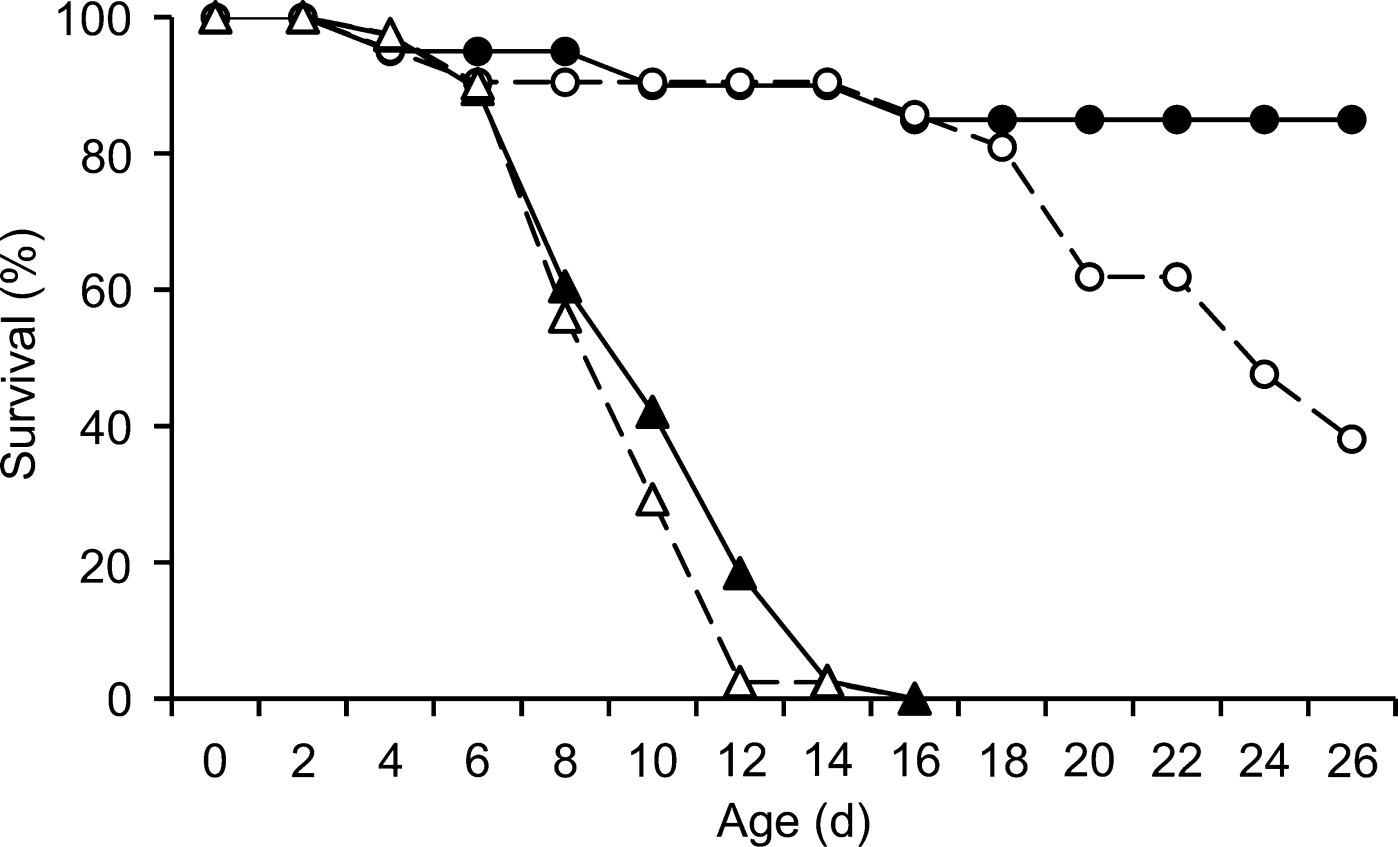
Percent of *Daphnia magna* hosts surviving over time between P-sufficient (filled dots), infected P-sufficient (open dots), P-limited (filled triangles), and infected P-limited (open triangles) treatments.

According to Cox regression, survival of *Daphnia* consuming P-limited food was lower compared with animals fed with P-sufficient food (Fig. 5, Table 1). The effect of infection alone was not statistically significant. However, as indicated by significant interaction between food and infection, and the negative coefficient estimate in Cox regression, infection decreased survival proportionally less within P-limited animals than within sufficient ones (Table 1).

**Table 1:** Coefficient estimates (B), SE values, and test statistics for terms in the final model and terms excluded from the model produced by Cox regression survival analysis

Terms in final model	B	SE	Wald	df	*P*-value	Exp(B)
Food	4.438	0.463	91.966	1	0.000	84.597
Time*Food*Infection	0.054	0.016	11.544	1	0.001	1.056
Food*Infection	−1.35	0.626	4.614	1	0.032	0.260
Terms not included in final model			Score	df	*P*-value	
Infection			0.141	1	0.707	

Exp(B) is the associated mortality risk, that is, a value greater than 1 indicates an increased mortality risk compared with the baseline group, and a value less than 1 indicates a lower mortality risk. The survival of P-limited animals is contrasted against P-sufficient individuals. In interaction terms, the infected animals are contrasted against uninfected ones within food treatments (and in time).

**Figure 6:**
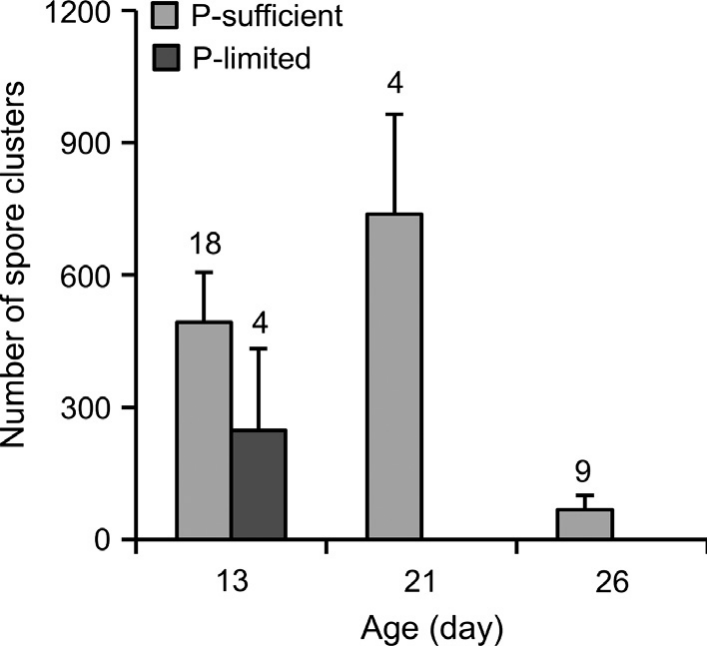
Mean (±SE) spore load in infected P-sufficient and P-limited *Daphnia magna* individuals. Sample sizes (number of *Daphnia* measured) are indicated above the bars.

In P-limited animals, few spore clusters were visible in two of the five animals dissected at day 9. At day 13, the mean number of spores did not differ statistically significantly between P-limited and P-sufficient animals (Mann–Whitney *U*-test, *U* = 22, *P* = 0.26, Fig. 6). Due to low survival, the development of the spore load of P-limited animals could not be followed further. In *Daphnia* consuming P-sufficient food, the spore load remained the same between days 13 and 21 (Kruskal–Wallis *H*-test; pairwise comparisons: ages 13 and 21 days, *z* = −0.99, *P* = 0.97, Fig. 6). However, at the end of the experiment (day 26) the remaining animals had significantly lower spore load as compared with day 21 (*χ* = 10.4, df = 3, *P* = 0.02, pairwise comparisons for ages 21 and 26 days, *z* = 15.1, *P* = 0.03).

**Figure 7:**
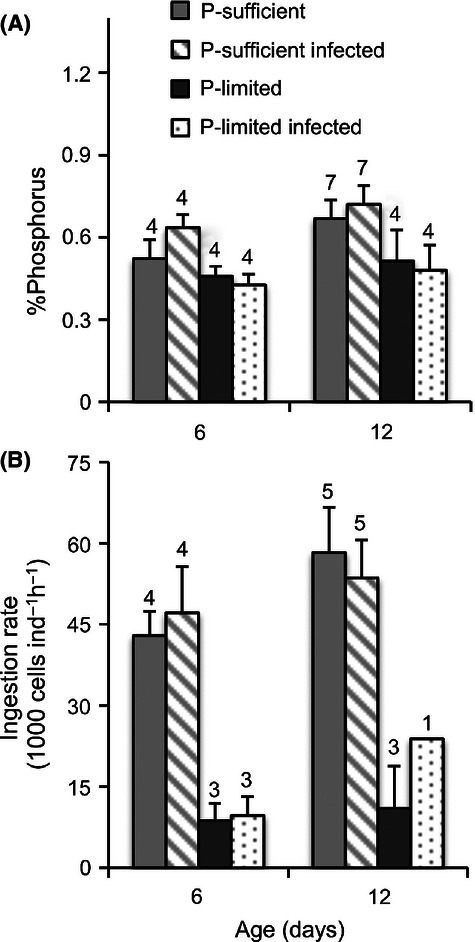
Mean (±SE) body P content (A) and ingestion rate (B) in response to infection status and food quality. Sample sizes (number of replicates measured) are indicated above the bars.

P-limited *Daphnia* had lower body P content than P-sufficient animals (three-way ANOVA, F_1,30_ = 19.9, *P* < 0.001, Fig. 7A). Juvenile (6 days old) animals had less phosphorus than mature (12 days old) animals (F_1,30_ = 4.2, *P* = 0.05). P-limited *Daphnia* had lower ingestion rate than P-sufficient animals (three-way ANOVA, F_1,20_ = 38.6, *P* < 0.001, Fig. 7B). Neither body P content nor ingestion rate differed between uninfected and infected animals.

## Discussion

The negative effects of both P-limitation and infection with *G. intestinalis* on *Daphnia* growth and survival found in this study agree with previous findings (e.g., Sundbom and Vrede 1997; Urabe et al. 1997; Ebert et al. 2000; DeMott 2003; Acharya et al. 2004; Jeyasingh et al. 2011). However, when we combined both stressors, we found that juvenile growth rate increased as compared with uninfected animals under severe P-stress. All P-limited individuals infected or not, died before day 16, being not able to reproduce before death. However, the parasite was able to drain enough energy from host to develop and produce spores even under extreme P-limitation, as there was no statistical difference in the number of spore clusters detected in P-limited or P-sufficient individuals by day 13.

Threshold elemental ratio (TER, the minimum needed for somatic maintenance) of C:P (atomic ratio) is suggested to be around ∼250 for *Daphnia* (Sterner and Hessen 1994; Elser et al. 2000) and thus our P-deficient treatment (C:P ∼ 1000) simulated extreme P-limitation. This resulted in physiological impairments such as molting disruption, with old molts remaining attached to posterior carapace as described previously by Sterner et al. (1993). The decrease in growth efficiency under P-limitation was consistent with the growth rate hypothesis, which states a positive association of body P content with rRNA content and protein synthesis rate, and thus with the growth rate of an animal (Elser et al. 2003). We also found that the ingestion rate and thus food input for the P-limited *Daphnia* was lower than for the P-sufficient animals, which corresponds to the results of He and Wang (2008).

Interestingly, infected animals had higher growth rate than uninfected ones under P-limitation. This could be due to differences in feeding behavior, but we found corresponding ingestion rates in infected and uninfected animals. As part of the body P content of infected animals was incorporated into the parasite, which could not be separated from the host during the P-analysis, and the P contents in uninfected and infected animals were similar, infected animals could have been expected to be even more P-limited in their growth than uninfected ones. Thus, we consider it unlikely that differences in growth rate were due to P-metabolism either. While we cannot rule out the possibility of parasite causing changes in the metabolism of other nutrients important for growth (e.g., nitrogen; Sterner and Elser 2002) that we did not manipulate in the experiment, we suggest that our results are due to the parasite causing changes in carbon (C) allocation of the hosts. The parasite we used, *G. intestinalis,* is a microsporidian, which are known to infect particularly fat tissues of hosts (Wittner and Weiss 1999). Microsporidians do not have their own energy metabolism, but instead exploit host lipid and glucose storages for spore construction, thus being dependent on the host for C (Biderre et al. 2000; Rivero et al. 2007). Under extreme P-limitation, *Daphnia* have to cope with large amounts of excess C in order to maintain somatic stoichiometry. *Daphnia* store some amount of excess C as lipids (Tessier et al. 1983; Sterner et al. 1992), but most of the leftover C is actively disposed via increased respiration and excretion of dissolved organic carbon (DOC) (Darchambeau et al. 2003; Anderson et al. 2005; Jensen and Hessen 2007; He and Wang 2008; Hessen and Anderson 2008). Our hypothesis is that due to exploitation of lipid storage by the parasite, infected *Daphnia* was able to convert a larger amount of excess C into lipids, and possibly also had decreased costs of excreting the extra C. However, after a longer P-limitation, the mismatch between the gain and physiological demands for P led to a premature death of *Daphnia*.

In P-sufficient *Daphnia*, parasite infection impaired growth, which was further reflected as a lower body mass at maturity and lower overall reproductive output as compared with uninfected animals. Similar to P-limited animals, no difference in ingestion rate or body P content between uninfected and infected individuals was detected. While the decrease in growth rate in infected animals might have been due to drainage of P for parasite growth, this is not supported by the opposite results in P-limited animals. However, also this result is consistent with parasite exploiting host lipids and diminishing C available for host somatic growth. We did not measure lipid contents of our experimental animals, but *Daphnia* body mass and visual lipid content are known to correlate positively (Tessier et al. 1983).

Under sufficient P-supply, spore loads were high between days 13 and 21 and then rapidly declined. Although *G. intestinalis* is considered as a rather benign parasite (Ebert et al. 2000), it produces spores constantly and the number of infected gut cells increases with host age (Ebert 1994, 1995). The decline in the spore loads at the end of the experiment might have been due to death of individuals with the highest spore loads. Individual differences in exposure to parasite at the beginning of the experiment could have subsequently led to differences in the spore load and in the timing of the parasite-induced host death, the risk being highest in hosts with higher spore loads (Ebert 1995).

Several virulent *Daphnia* parasites, for example, *Pasteuria ramosa* (Ebert et al. 2000; Frost et al. 2008), *Polycaryum laeve* (Johnson et al. 2006b; Forshay et al. 2008), and *Metschnikowia bicuspidata* (Ebert et al. 2000; Hall et al. 2009) have been suggested to be nutrient limited (Forshay et al. 2008; Frost et al. 2008; Civitello et al. 2012). On the other hand, several microsporidian parasites, for example, *Hamiltosporidium tvaerminnensis* (formerly *Octosporea bayeri;* Haag et al. 2011), *H. magnivora* (formerly *Flabelliforma magnivora*; Haag et al. 2011), as well as *G. intestinalis,* are rather avirulent to *Daphnia* hosts (Ebert 1995, 2005). Apart from differences in transmission modes, possibly also lower dependence on other elements than carbon of *Daphnia* by the parasite might facilitate the less destructive exploitation of the host. The connections between parasite virulence and their stoichiometric demands would, however, need more experimental evidence.

Our results suggest that the impact of the stressor pair composed of parasite infection and low food nutrient concentration might depend on the nutrient requirements of the parasite in question. During the last decade, the significant role of parasites in food webs and ecosystems has been acknowledged (Lafferty et al. 2008; Amundsen et al. 2009). Only few studies have reported interactions between parasite infection dynamics and nutrients in nature (Johnson et al. 2007; Aalto et al. 2012; Civitello et al. 2012). However, in order to reliably predict the role of parasites in food webs, further knowledge on host–parasite interactions under different nutrient regimes is needed.
